# An Eye for an Eye? Third Parties’ Silence Reactions to Peer Abusive Supervision: The Mediating Role of Workplace Anxiety, and the Moderating Role of Core Self-Evaluation

**DOI:** 10.3390/ijerph16245027

**Published:** 2019-12-10

**Authors:** Jun Huang, Gengxuan Guo, Dingping Tang, Tianyuan Liu, Liang Tan

**Affiliations:** 1 School of Economics and Management, Southwest University, Tiansheng Road2#, Chongqing 400715, China; john0277@swu.edu.cn (J.H.); tang19960125@email.swu.edu.cn (D.T.); 2 School of Sociology, Wuhan University, 299 Bayi Road, Wuhan 430072, China; ltyuan@whu.edu.cn; 3 School of International Business, Sichuan International Studies University, No.33 Zhuangzhi Road, Chongqing 400031, China

**Keywords:** affective events theory, peer abusive supervision, workplace anxiety, silence

## Abstract

Currently, a few scholars have studied the spillover effects of abusive supervision from third parties’ perspective. However, these limited researches mainly focus on third parties’ explicit behavior response to peer abusive supervision, ignoring their implicit reactions (e.g., silence) and the emotional mechanism among it. To fill the above gaps, drawing on affective events theory, we construct a theoretical model that explains the relationship among peer abusive supervision, third parties’ workplace anxiety, third parties’ silence, and third parties’ core self-evaluation. Multi-wave data from 283 front-line employees (57% male and 43% female; 57.2% are 30 years old and below, 31.1% are 31–40 years old and 11.7% are over 40 years old), who come from eight real estate and insurance companies in China, were used to support our framework. In particular, our empirical results indicated that peer abusive supervision was positively related to third parties’ silence, among which workplace anxiety played a partial mediating role. In addition, third parties’ core self-evaluation moderated the relationship between peer abusive supervision and silence, meanwhile, the mediating role of workplace anxiety. Specifically, the effect of peer abusive supervision on workplace anxiety, and the mediating effect of workplace anxiety, was weaker when the third parties’ core self-evaluation was higher rather than lower. The results contribute to both theory and practice.

## 1. Introduction

Abusive supervision can be manifested in ridicule, public humiliation, and deliberate indifference, etc. It is defined as “subordinates’ perceptions of the extent to which supervisors engage in the sustained display of hostile verbal and nonverbal behaviors, excluding physical contact” [1]. The existing studies on the consequences of abusive supervision focus on its effects on the abusive supervisors and abused subordinates. For instance, a large body of literatures have shown that abusive supervision increases employees’ psychological distress, workplace deviance, and decreases their creativity and task performance [2,3]. However, in organizational practice, abusive supervision is not only related to the above two parties. Furthermore, it also involves third parties (coworkers of abused subordinates) in the same workplace, whereby it may have an impact on third parties’ emotions and behaviors. Peer abusive supervision refers, from the perspective of third parties, to the abusive behavior of supervisors towards coworkers [4]. At present, a few scholars have explored the impact of peer abusive supervision on third parties. When third parties perceive peer abusive supervision they generate a supervisor-directed deviance, and this reduces work performance among other factors [5,6].

Nevertheless, the limited literatures mainly explore the influence of peer abusive supervision on the explicit behavior of third parties, which ignore the research on implicit behavior such as silence in the workplace. Thus, when people make decisions, they prioritize the consequences and gains compared with weighing the probability of things happening [7]. When third parties respond to peer abusive supervision through explicit behaviors such as counter-work behavior and organizational deviant behavior, they are easily subject to the rules and regulations of the organization, or even suffer retaliation from supervisors. These above carry a higher risk [8,9]. In other words, the existing literatures fail to consider that when third parties respond to peer abusive supervision, the possible self-protection motivation in their behavioral choices. Silence has the characteristic that it is not easy to be perceived, so the possibility of getting punishment from the organization is greatly reduced. This conforms to the risk assessment of third parties. Previous studies have found that silence not only leads to decision-making mistakes, but also damages the positive atmosphere within an organization [10,11]. For example, silence often obstructs the learning and development process of an organization, which can prevent internal and external stakeholders from harm [12,13]; Similarly, MacKenzie and his colleagues argue that employees’ silence destroys the climate for voice within the group that encourage others to speak up, which further negatively influence the team performance [14]. However, there are a lack of empirical studies on the impact of peer abusive supervision on employees’ silence from the perspective of third parties. 

In addition, Dhanani and LaPalme pointed out, in the review literature, that although some empirical studies have examined the negative impact of peer abusive supervision on the behavior of third parties, unfortunately, very few studies have focused on the mediating mechanisms of third parties’ emotions in the above process [15]. It is important to note; however, that emotions play a vital role during the processes through which negative attitudinal outcomes are produced [16]. Therefore, it is high time for the exploration of the emotional mechanism, explaining the negative spillover effect of the dark-side leadership to third parties’ behavior, which helps to open the “black box” between peer abusive supervision and third parties’ silence.

Therefore, to fill these gaps, drawing on affective events theory [17], this paper constructs a theoretical framework to explore the impact of peer abusive supervision on third parties’ silence, and the mechanisms among it, together with the boundary conditions for the influences of peer abusive supervision on third parties. Affective events theory pays attention to the impact of work events on emotions and the further effect on individual’s attitudes and behaviors. The theory suggests that the work events experienced by employees will trigger positive or negative emotional experiences of employees, and emotions will further affect their attitudes and behaviors [18]. Specifically, we suggest that when witnessing peer abusive supervision, third parties will evaluate the work event and their own resources. Then, they will take a silent way to respond to the unfair phenomenon in the organization from the motivation of self-protection. Furthermore, the emotions generated in the cognitive assessment process influence their subsequent behavioral reactions [19]. Thus, anxiety plays a key role in the relationship between work stress and employees’ behavioral reactions [20]. Hence, we further examine the emotional mediating mechanism of third parties’ workplace anxiety between peer abusive supervision and silence. Finally, according to the affective events theory, employees’ emotional reaction to work events can also be affected by individual traits [17]. The paper argues that the higher the core self-evaluation of third parties, the weaker the emotional mediating effect of workplace anxiety.

In conclusion, we expect to make theoretical contributions from the following aspects. First, the present paper is a timely response to the call that prior researches on the theme of abusive supervision have a single perspective, and researchers need to pay attention to study the impact of abusive supervision from multiple perspectives [6,21,22]. This is conducive to enriching the traditional literature on the consequences of abusive supervision, which are mainly concentrated on bilateral perspectives. Second, this is different from the prior literatures, which mostly focus on exploring the impact of peer abusive supervision on the explicit behavior of third parties. Based on the research framework of affective events theory, we discuss the possible self-protection motivation of third parties in the process of evaluating workplace events. It lays a cognitive foundation for the choice of the implicit behavior of silence of the subsequent third parties. Furthermore, this paper responds to the call for paying attention to the mediating role of the emotional reactions of third parties, between peer abusive supervision and their behavioral choice [15]. Drawing affective events theory, we construct a model of emotional mechanism between peer abusive supervision and third parties’ silence, and suggest that peer abusive supervision affects silent behavior indirectly by triggering workplace anxiety. Finally, by introducing core self-evaluation as a moderating variable to verify the boundary condition of peer abusive supervision’s spillover effect, it further confirms when our mediation mechanism will work. 

**Figure 1:**
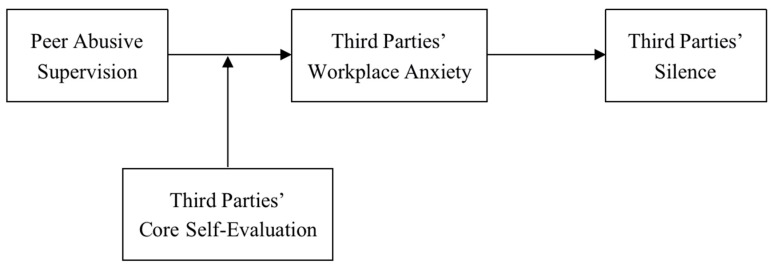
The theoretical framework.

Our theoretical framework is presented in Figure 1.

## 2. Theory and Hypotheses 

### 2.1. Affective Events Theory

Affective events theory (AET) refers to work events (e.g., peer abusive supervision) experienced by employees that elicit an emotional experience. The accumulation of emotions further affects an employees’ attitude and behavior [17]. AET divides the evaluation of work events into two processes: primary appraisal and secondary appraisal [23]. Primary appraisal only focuses on whether the event is consistent with their own goals, values, or conflicts, and whether the event is beneficial to themselves. Secondary appraisal has a more meaningful analysis of the event, such as evaluating whether the individual has sufficient resources to deal with the event. Individual emotions arise from the secondary appraisal process. In particular, not all work events induce emotional reactions. For example, some mild events are not related to the individual’s own goals and values. Furthermore, the evaluation of such events only stays at primary appraisal stage, without secondary appraisal. Thus, it does not induce any emotional reactions [16,24,25].

Emotional state is the basis of individuals’ thinking and behavior in work [26,27]. There are two main ways for emotional experience to affect individual behavior: One is that emotion can directly affect employee’s behavior and generate affect-driven behaviors; the other is that emotion can also affect an employee’s cognition and judgment. It will then affect the employee’s behavior, and produce judgment-driven behaviors. According to AET, work environment factors, (such as work characteristics and organizational atmosphere, etc.), can affect employees’ experience of work events. These will lead to positive and negative emotions that affect individual behaviors and attitudes [20,28,29]. Individual traits also contribute to the formation of these positive and negative emotions [26].

### 2.2. Peer Abusive Supervision and Third Parties’ Silence

AET regards work events as the inducing factors of individual emotional reactions. These emotional events are of importance to employees, and can come from either the work itself (e.g., increased workload) or interpersonal interactions in the workplace (e.g., peer abusive supervision). Third parties share the same working environment with their supervisors and abused subordinates. Thus, the interpersonal interaction between supervisors and subordinates is of great importance to third parties. We suggest that this interpersonal interaction also constitutes the emotional events of third parties. According to AET, employees’ evaluation of work events are divided into two processes: primary appraisal and secondary appraisal [23]. We demonstrate it based on this basic assumption.

When witnessing peer abusive supervision, third parties first make a primary appraisal of the work event. They then judge whether it is consistent with their own values and interests [17]. In the workplace, due to the existence of professional ethics and morality, supervisors and subordinates work together through cooperation to complete work-related tasks. Ridicule, public humiliation, and deliberate indifference shown by abusive supervision violate the ethics of workplace [1] and destroy the harmonious work atmosphere. As such, when witnessing peer abusive supervision, third parties will sympathize with the abused coworkers, question the professional norms in their workplace, and doubt their own future treatment [30]. They cannot help asking themselves: "Will I be the next employee to be abused?” In response to this type of organizational injustice, third parties may be reluctant to actively pursue work for the benefit of the organization, and they may take a more negative attitude and behavior towards their organization. Consequently, individuals may be indifferent to the problems existing in the organization, or even show schadenfreude. In contrast, maintaining silence in the workplace is a relatively safe way [31]. Thus, silence becomes a direct choice for individuals to face peer abusive supervision.

Second, third parties make a more meaningful analysis of peer abusive supervision, such as assessing whether they have enough resources to deal with the incident. Previous studies have shown that negative interpersonal experience is an important source of workplace stress for employees [32,33]. Therefore, we suggest that peer abusive supervision constitutes a source of stress for third parties at the same time. When third parties are under pressure, they need to spend time and energy to cope with the pressure and control their emotions, rather than focusing on finding work problems, making suggestions, and solutions. On the other hand, the pressure consumes a large amount of resources from third parties. In this way, they may be more inclined to keep silent to protect their resources owing to their own interests. This is because voice is usually costly and risky, and it is highly likely to consume the remaining resources of the third parties [34,35].

In summary, we propose hypothesis 1:

**Hypothesis 1** **(H1):** *Peer abusive supervision is positively related to third parties’ silence.*

### 2.3. The Mediating Role of Third Parties’ Workplace Anxiety

Based on AET, the emotional reactions of third parties are generated in secondary appraisal [23]. Thus, not all work events can induce emotional reactions, such as some mild events that are not related to an individual’s own goals and values. Indeed, the evaluation of such events may only stay at primary appraisal, without a secondary appraisal. As such, it does not induce emotional reactions [16,24,25]. However, deliberate indifference, ridicule, laughing, and other behaviors in the form of abusive supervision destroy ethics in the workplace. In this way, this negative work event is not mild, on the contrary, it stimulates the third parties’ secondary appraisal and triggers their emotional reactions. 

Workplace anxiety refers to the employees’ feeling of nervousness and worry about completing work tasks [36]. It is the tension and worry that individuals feel when they face potential threats. It represents the stress response of employees to symptoms of tension [37,38]. Workplace anxiety is a state emotion in the workplace. It usually occurs when individuals are under pressure, or are in an uncertain organizational environment. 

According to AET, in the process of secondary appraisal, when third parties face the pressure source of peer abusive supervision, they may first assess the threat of the event or situation to themselves, and subsequently assess whether they have sufficient resources to deal with the work event [17,39]. When third parties perceive that there is a threat to their own goals or interests in the workplace, they often have an uncertain perception about their work environment, and will bring some negative emotional reactions. Anxiety acts as a self-signal when individuals’ survival is threatened, which is often accompanied by a threat [40]. Hence, we speculate that peer abusive supervision perceived by third parties affects their workplace anxiety level. When they lack assurance, the third parties tend to generate workplace anxiety [41,42]. 

Given these rationales, we propose hypothesis 2:

**Hypothesis 2** **(H2):** *Peer abusive supervision is positively related to third parties’ workplace anxiety.*

Emotions in the process of cognitive assessment affect their subsequent behavioral reactions [19]. Therefore, anxiety may play a key role in the relationship between work pressure and employee behavioral responses [20]. As previously mentioned, there are mainly two ways in which emotional experience can affect individual behaviors: One is that emotions can directly affect the employees’ behaviors and generate affect-driven behaviors; the other is that emotions can also affect the employees’ cognition and judgment. This in turn affects their behaviors, and leads to judgment-driven behaviors. Based on AET, we discuss the above basic assumptions as follows:

The first is the affect-driven behaviors path. For third parties, workplace anxiety is a negative emotion that stimulates individuals to take action to escape from an anxiety-inducing situation [43]. Third parties who are anxious will generate psychological insecurity, and will further stimulate their silence [44]. 

The second is the judgment-driven behaviors path. In the workplace, supervisors have the power on important work-related issues, such as job assignments, performance measurement, etc. [45]. Voice is also a high-risk activity. If it cannot be recognized or accepted, it often leads to negative performance evaluations, unfavorable work arrangements, and even severe negative results, such as retaliation [46]. Therefore, third parties can also evaluate the safety of the behavior before voicing. However, anxiety and subsequent threats affect the information processing of third parties. This leads them to tend to choose threatening information, and thus interpret and deal with threatening events, in an ambiguous way. This will affect the individual’s moral judgment and behavior. It makes them ignore the ethical behavior [47,48]. Thus, due to excessive consumption of emotional resources, third parties with workplace anxieties will find it difficult to make an accurate assessment of the expected behavior choice. For their own safety, third parties often choose silence in response to peer abusive supervision. Thus, the study proposes that peer abusive supervision leads to third parties’ workplace anxiety. Consequently, in order to alleviate the impact of such pressure source and escape from anxiety, individuals choose silence for self-protective motivation. 

To sum up, we hypothesize the following:

**Hypothesis 3** **(H3):** *Third parties’ workplace anxiety is positively related to their silence.*

**Hypothesis 4** **(H4):** *Third parties’ workplace anxiety plays a partial mediating role between peer abusive supervision and their silence.*

### 2.4. The Moderating Role of Third Parties’ Core Self-Evaluation

Core self-evaluation is the most basic evaluation of an individual’s own ability and value. It is a potential and broad personality structure, covering four dimensions: self-esteem, self-efficacy, neuroticism, and control points [49]. Individual characteristics of employees are important factors that affect their behavior selection and adaptation to work events. Third parties with a high level of core self-evaluation form positive self-schema in their minds. Thus, individuals have a certain positive evaluation tendency on the work events they have experienced [50]. For instance, individuals with high core self-evaluation accept challenging work, and consider that their work is valuable [51]. Based on affective events theory, this is directly related to the secondary appraisal effect of emotional cognitive evaluation. In the secondary appraisal stage, if third parties show a positive evaluation tendency towards their perceived work events (peer abusive supervision), then the individual perceives that they have enough resources to deal with the pressure source. Subsequently, the negative effects of the peer abusive supervision on individual emotional and psychological problems will be reduced. In other words, third parties with a high core self-evaluation hold a positive view of peer abusive supervision, and believe that they have sufficient ability and resources to deal with this work event. In this respect, they do not need to invest more resources in emotional regulation. Thus, the impact of peer abusive supervision on third parties’ workplace anxiety is weakened. For third parties with a low core self-evaluation, they may have a negative evaluation tendency towards peer abusive supervision. Previous studies have also shown that employees with a low core self-evaluation are often under pressure [52]. Therefore, in this case, individuals tend to invest too much resources to deal with the pressure source. This may aggravate third parties’ workplace anxiety on peer abusive supervision. 

Therefore, we propose the following:

**Hypothesis 5** **(H5):** *Third parties’ core self-evaluation moderates the relationship between peer abusive supervision and third parties’ workplace anxiety, such that the relationship is weaker when third parties’ core self-evaluation is higher rather than lower.*

In line with the logic of the above hypothesis and the relevant studies on mediating and moderating effects, this study further proposes a moderated mediation model [53]. It means that the mediating effect of workplace anxiety changes in relation to different levels of third parties’ core self-evaluation. Specifically, third parties with a high core self-evaluation will weaken the positive impact of the peer abusive supervision on silence through workplace anxiety; In contrast, third parties with low core self-evaluation will strengthen the positive impact of peer abusive supervision on silence through workplace anxiety.

Therefore, we propose the following:

**Hypothesis 6** **(H6):** *Third parties’ core self-evaluation moderates the mediating effect of third parties’ workplace anxiety on the relationship between peer abusive supervision and the third parties’ silence, to an extent that the mediating effect is weaker when third parties’ core self-evaluation is higher rather than lower.*

## 3. Methods

### 3.1. Samples and Procedure

Considering the overarching theory of our research is the affective events theory, an event-based within-person framework was used, which means our model should be tested at the individual level [29,54]. In order to avoid violating the assumption of independence in our data and to perform a multi-wave study, following the previous studies using affective events theory, we collected our samples randomly without forming the multiple subordinates nested [55,56]. Data for this study were collected from eight real estate and insurance companies in China, and participants in the survey were all front-line staff. Each of the above companies had over 50,000 employees. With the help of the human resource departments of these companies, the questionnaires were distributed randomly by these companies’ e-mail systems. All of the variables were measured by employees’ self-report. The whole research process followed: First, the research team communicated with the human resource directors of the eight enterprises to determine the investigation scheme. Second, 350 employees were randomly selected by the research team according to the list of employees provided by these enterprises. Finally, in order to avoid common method variance, the research team adopted a psychological separation method to set the questionnaire items, together with using a time lag method, similar to previous studies, which means we measured our variables at different times [57,58,59].

The researchers collected data at multi-wave time points as follows: At time 1, participants were asked to evaluate the perceived phenomenon of supervisors abusing their coworkers. A total of 350 questionnaires were distributed, and 317 were effective. These were taken back for analysis. Two weeks later, at time 2, 317 effective questionnaires were measured again. This mainly asked participants to evaluate their workplace anxiety and silence. A total of 317 questionnaires were distributed, and 303 effective questionnaires were received. Then two weeks later, at time 3, the questionnaire survey was conducted again for the 303 employees that had filled in valid questionnaires. Participants were mainly asked to evaluate their core self-evaluation in order to understand the basic information of participants; 283 valid data were obtained. Demographic information is shown in Table 1.

### 3.2. Measures

The study adopted the Likert five-item scale to measure the above dimensions. We mainly refer to the authoritative scales. The words were modified based on the pre-investigation.

#### 3.2.1. Peer Abusive Supervision

To measure peer abusive supervision perceived by third parties, we adopted the five-item abusive supervision scale adapted from Mitchell and Ambrose by Ping et al. A sample item was "My supervisor said that my coworker’s thoughts were stupid" [60,61]. The Cronbach’s Alpha for this scale was 0.940.

#### 3.2.2. Workplace Anxiety

To measure third parties’ workplace anxiety, we adopted the four-item scale developed by Jonge and Schaufeli for reference [62]. A sample item was "I am afraid that my work performance is worse than others", and the Cronbach’s Alpha was 0.902.

#### 3.2.3. Silence

We used the five-item scale developed by Tangirala and Ramanujam to measure the third parties’ silence [63]. A sample item was "I do not tell others about potential problems in the company I noticed", the Cronbach’s Alpha was 0.946.

#### 3.2.4. Core Self-Evaluation

We adopted the twelve-item scale developed by Judge et al. [64] to measure third parties’ core self-evaluation. A sample item of the scale was "When I try hard, I generally succeed", the Cronbach’s Alpha was 0.972.

#### 3.2.5. Control Variables

In addition, some studies have shown that employee’ backgrounds (such as age, gender, education level, etc.) are also important factors affecting behavior of third parties. Therefore, this study had taken gender (1 for male, 2 for female), age (1 for participants 30 years old and below, 2 for participants 31–40 years old, 3 for participants over 40 years old), education level (1 for senior high school and below, 2 for training school, 3 for undergraduate, 4 for postgraduate and above) as control variables. These questions were completed by staff.

## 4. Results

### 4.1. Common Method Variance

In order to control for common method variance, the data in this paper was anonymized in the collection stage. It considers that the core variables involved in peer abusive supervision, third parties’ workplace anxiety, silence, and core self-evaluation are all evaluated by third parties themselves. Thus, the relationship between these variables may still be affected by the common method variance. To test it, we used principal component analysis to analyze the items of all questionnaires, and separated the common factors that were not rotated. The results showed that there were six factors more than 1. The first factor accounted for 32.106%, and the total interpretation variance was 79.891%, so it met the requirement that the maximum extraction variance should be less than 50% critical value of the total interpretation variance. This indicated that common method variance in this study is in an acceptable range and can be statistically analyzed.

### 4.2. Confirmatory Factor Analysis (CFA)

Based on the four core variables, namely peer abusive supervision, workplace anxiety, silence, and core self-evaluation of third parties, we constructed five models. AMOS 24 was adopted to verify the confirmatory factor analysis on these models, and to test the structural validity of the scale from the perspective of discriminant validity. According to the comparison among the single-factor model, the two-factor model, three-factor model, and four-factor model, we found that the four-factor model has a better fit. Thus, the fit of this model was better than other models. For details see Table 2. This showed that the four core constructs of this study (peer abusive supervision, workplace anxiety, silence, and core self-evaluation) all had a good discrimination validity.

### 4.3. Correlation Analysis

According to the data presented in Table 3, we found that peer abusive supervision was significantly and positively related to third parties’ workplace anxiety (r = 0.304, *p* < 0.01). Peer abusive supervision was significantly and positively related to the silence of third parties (r = 0.382, *p* < 0.01), and workplace anxiety was significantly and positively related to silence (r = 0.351, *p* < 0.01). Therefore, the above results concerning the correlation between variables were essentially consistent with the research hypotheses. In this way, we used multistage regression analysis further.

### 4.4. Hypothesis Testing

In order to test the impact of peer abusive supervision on third parties’ silence, together with the mediating role of workplace anxiety and the moderated mediation role of third parties’ core self-evaluation, we first used SPSS 24.0 to conduct a multistage regression on the research data collected by the research group. Next, we conducted a bootstrap analysis using Hayes’ PROCESS for SPSS to test the robustness of our model [65].

First, the main effects, which examined the impact of peer abusive supervision on third parties’ silence. When third parties’ silence was set as the dependent variable, model 1 was the regression model of the control variable to third parties’ silence, and model 2 was the regression model of third parties’ silence after adding the independent variable (peer abusive supervision). According to Table 4, peer abusive supervision was significantly and positively related to silence in model 2 (β = 0.402, *p* < 0.01). Thus, hypothesis 1 was supported.

Second, the mediation effects. Specifically, whether the mediating effect of workplace anxiety on the relationship between peer abusive supervision and third parties’ silence exists was explored further. According to Table 5, peer abusive supervision had a significant positive effect on third parties’ workplace anxiety in model 9, (β = 0.315, *p* < 0.01) and in this way, hypothesis 2 was supported. Meanwhile, in model 3, the third parties’ workplace anxiety had a significant positive effect on silence (β = 0.326, *p* < 0.01) and hypothesis 3 was supported. Adding the mediator variables (workplace anxiety), in model 4, although the effect of peer abusive supervision on silence of third parties (β = 0. 333, *p* < 0.01) was lower than model 2 (β = 0.402, *p* < 0.01), it was still significant. In addition, the third parties’ workplace anxiety still had a significant positive effect on silence (β = 0.221, *p* < 0.01). Therefore, these findings were preliminary evidences for hypothesis 4. In order to further verify the mediation effect of third parties’ workplace anxiety between peer abusive supervision and third parties’ silence, we used the PROCESS module to analyze the significance of mediating effect. The results presented in Table 6 indicate that a 95% bias-corrected bootstrap confidence interval was [0.0311,0.1706] (excluding zero). Namely, the mediation effect of third parties’ workplace anxiety between peer abusive supervision and silence was significant. Thus, Hypothesis 4 was further supported.

**Figure 2:**
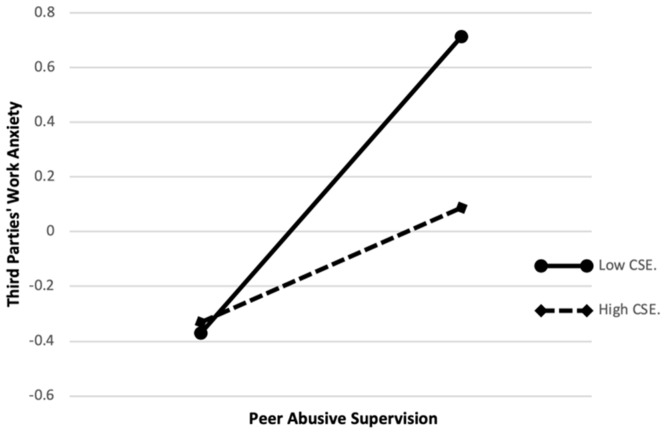
The moderating role of CSE.

Subsequently, the moderation effects verified whether third parties’ core self-evaluation has the moderating effect between peer abusive supervision and third parties’ workplace anxiety; meanwhile, whether third parties’ core self-evaluation can moderate the mediating effect of workplace anxiety on the relationship between peer abusive supervision and third parties’ silence. For eliminating the collinearity deviation, we standardized the four core variables that were involved in this paper, and used the standardized variables to compute the interaction terms. The results in Table 5 showed that in model 10, after adding the moderator (core self-evaluation), peer abusive supervision still had a significant positive effect on third parties’ workplace anxiety (β = 0.318, *p* < 0.01), while in model 11, when peer abusive supervision interacted with third parties’ core self-evaluation, the interaction was negatively and significantly related to workplace anxiety (β = –0.212, *p* < 0.01). In this respect, it indicated that the higher the third parties’ core self-evaluation, the weaker the positive effect of peer abusive supervision on workplace anxiety. Thus, hypothesis 5 was supported. In order to further explain the moderating effect of third parties’ core self-evaluation, we plotted the moderation effect chart in Figure 2. There was a stronger negative relationship between peer abusive supervision and workplace anxiety when third parties’ core self-evaluation was higher, compared to when it was lower.

Finally, the moderated mediation effects verified whether third parties’ core self-evaluation can moderate the mediating role of third parties’ workplace anxiety on the relationship between peer abusive supervision and silence. First, in model 5, adding third parties’ core self-evaluation to the regression model, the coefficient of peer abusive supervision (β = 0.406, *p* < 0.01) was significant. Then, the mediator (third parties’ workplace anxiety) was added into model 6. The mediator coefficient (β = 0.197, *p* < 0.01) was significant, as well. Combined with models 10, 5, and 6, it demonstrated that the mediation effect of the moderated mediation model was significant. After the interaction term of workplace anxiety and core self-evaluation was added to model 7, the interaction had a significant negative effect on silence of third parties (β = −0.109, *p* < 0.05). This indicated that the higher the third parties’ core self-evaluation, the lower the mediating role of workplace anxiety. Thus, hypothesis 6 was initially supported.

**Figure 3:**
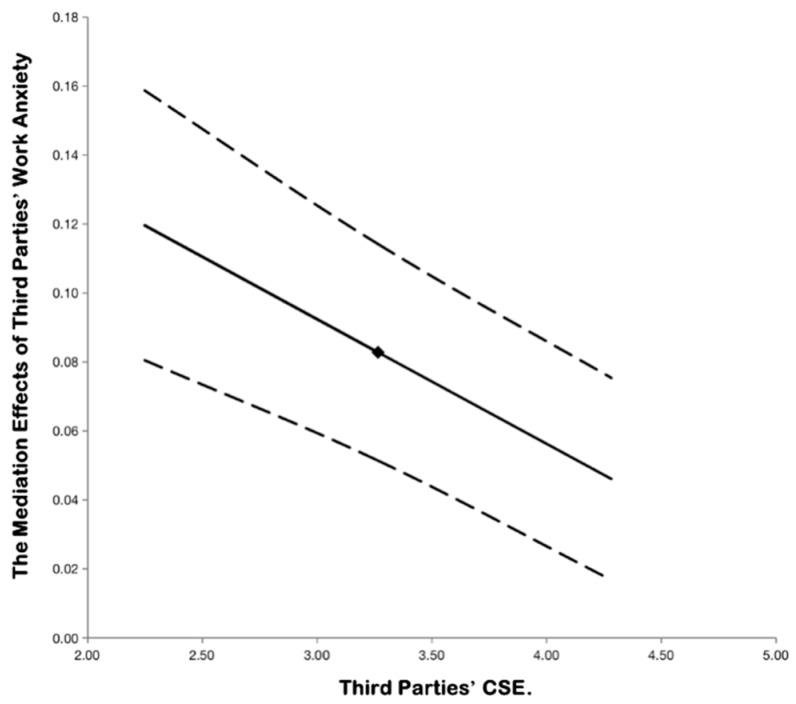
The moderated mediation role of CSE.

In order to further verify the proposed moderated mediation effects, we used the PROCESS module to further test whether third parties’ core self-evaluation can moderate the mediating role of third parties’ workplace anxiety. The results showed that the index of the moderated mediator is −0.0368, with a 95% confidence interval of [−0.0615, −0.0061], excluding zero. Combined with the results of Table 7, as third parties’ core self-evaluation varied from one standard deviation below the mean to one standard deviation above the mean, the mediating role of workplace anxiety decreases significantly. In other words, when the third parties’ core self-evaluation was at a lower level, the mediating role of workplace anxiety was stronger. Consequently, the moderated mediation effect proposed in the study was established, and hypothesis 6 was further verified. Referring to the interaction with mediators effect plotting method of Edwards and Schurer [66], the moderated mediator chart corresponding to hypothesis 6 is shown in Figure 3.

## 5. Discussion

All of our six hypotheses were verified. Peer abusive supervision is positively related to third parties’ workplace anxiety (H2) and silence (H1). Third parties’ workplace anxiety is positively related to their silence (H3), meanwhile, it plays a partial mediating role between peer abusive supervision and their silence (H4). In addition, third parties’ core self-evaluation moderates the relationship between peer abusive supervision and third parties’ workplace anxiety, such that the relationship is weaker when third parties’ core self-evaluation is higher rather than lower (H5). Further, third parties’ core self-evaluation moderates the mediating effect of third parties’ workplace anxiety on the relationship between peer abusive supervision and the third parties’ silence, to an extent that the mediating effect is weaker when third parties’ core self-evaluation is higher rather than lower (H6).

### 5.1. Theoretical Implications

First, the current study explores abusive supervision from the perspective of third parties, which enriches the research perspective on the consequences of abusive supervision. Our research focuses on peer abusive supervision, which is not only a beneficial supplement to the discussion of the consequences of abusive supervision from a third-party perspective, but also echoes prior studies criticizing the single perspective of abusive supervision, and appealing to the study of the impact of abusive supervision from multiple perspectives [6,21]. Our empirical results show that abusive supervision not only has direct negative effects on the abused subordinate, but also has negative spillover effects on third parties, which help researchers further confirm the dark-side outcome of abusive supervision from multiple perspectives. Thus, our framework does have a certain reference significance for understanding whether, when, and why peer abusive supervision affects third parties’ silence.

Second, the empirical results show that peer abusive supervision can also cause the implicit silence of third parties. At present, research on how third parties respond to mistreatment inflicting their coworkers is still at a nascent stage. Moreover, these limited researches mostly focus on the explicit behavior response of third parties to peer abusive supervision, such as organizational deviation, counter-work behavior, etc. [5]. They ignore the influence of third parties’ silence and other implicit behaviors. When third parties carry out explicit behaviors (such as counter-work behaviors and organizational deviant behaviors), they may suffer risks, and be vulnerable to retaliation from the organization’s regulations and supervisors [8,9]. Therefore, the existing literature ignores the self-protective motivation of third parties when witnessing peer abusive supervision. Skarlicki and Kulik argue that when third parties face inequalities such as bullying and exclusion in the workplace, their reactions to peer abuse may be contingent upon self-interest [67]. By analyzing the potential self-protective motivation of third parties in this process, the current study enriches the research on outcome factors of employees’ response to any mistreatment from the perspective of the third-party, which may provide us with deeper insight to explain workplace abusive phenomena. 

Third, Dhanani and LaPalme pointed out in the review literature that, although some empirical studies have examined the negative impact of peer abusive supervision on the attitude and behavior of third parties, few studies focus on the mediating mechanism of third parties’ emotions in the above process [15]. Hence, the current study is a timely response to the call of Dhanani and LaPalme. Drawing on affective events theory, we constructed a model on the emotional mechanism between peer abusive supervision and the behavioral response of third parties. The empirical results showed that peer abusive supervision further influences third parties’ silence by affecting their workplace anxiety. It has a certain reference value for the understanding of why peer abusive supervision causes silence among third parties. 

Finally, the present study empirically verifies the moderated mediation effects of third parties’ core self-evaluation on the main effect. Thus, third parties’ core self-evaluation not only alleviates the positive influence of peer abusive supervision on their workplace anxiety, but also reduces the mediating role of workplace anxiety on the main effect. The results have a certain guiding significance for us to understand the characteristics that third parties can effectively resist peer abusive supervision.

### 5.2. Practical Implications

The results of this study have important guiding significance for the practice of human resource management in organizations, which are embodied in the following three aspects. First, combined with previous literature, empirical results, and the study of abusive supervision, we found that in most cases, the implementation of abusive supervision by supervisors has a negative impact on organizations and individuals. These detrimental influences are not only related to both parties, but also affect the third parties in the workplace. When witnessing peer abusive supervision, the third parties evaluate the relevance of work events to themselves, and then respond to the unfair phenomenon in the organization, based on the self-protective motivation, by silence. However, the silence is purposeful, namely they deliberately hide their opinions and suggestions that can improve organizational performance [68]. This is obviously not conducive to the sustainable development of the organization. Hence, it is necessary to first launch regular management skills training to supervisors, improve their awareness of the harmfulness of abusive supervision, and minimize the possibility of supervisors adopting such negative behavior. Concurrently, in order to strengthen the third parties’ willingness to communicate for this phenomenon, an improved employee feedback mechanism should be established within enterprises. Employees should have a dominant position in the supervisor evaluation system, and then reduce the potential risks perceived by breaking silence or taking suggestions. This correspondingly increases the opportunity cost of abusive supervision. 

Second, the results indicate that peer abusive supervision triggers third parties’ workplace anxiety, and further promotes their silence, which may further also disrupt the organizational climate. Thus, abusive supervision, as a more frequent stress event in the workplace, for its uncertain perception, inevitably leads to the negative emotion of third parties. Therefore, in management practice, if because of the situation or other unavoidable circumstances, supervisors adopt abusive supervision, they should provide subordinates with more information sources for diagnosis. This will act as a way to form comprehensive cognitive judgment of the event, and reduce the possibility of third parties’ workplace anxiety. In addition, organizations should also set up a set of psychological counseling mechanisms and procedures for employees to provide a consultation platform, and safeguard measures for their emotional relief after experiencing peer abusive supervision. This will help to prevent the situation from worsening. On the basis of improving the mental health of third parties, organizations should reduce the harm of their subsequent silent behavior to the organization’s interests. 

Third, third parties’ core self-evaluation can not only depress the positive impact of peer abusive supervision on their workplace anxiety, but also reduce the mediating role of workplace anxiety in main effect. Therefore, in order to minimize the cost that enterprises pay to reduce the negative impact of abusive supervision, the most economical measure should strengthen the assessment of employees’ characteristics in the recruitment process. In this way, it will give priority to employees with strong confidence and who tend to make positive judgments on aspects, together with undertaking risk prevention and control in advance.

Finally, our empirical results have shown that abusive supervision not only has direct negative effects on abused subordinates, but also has negative spillover effects to third parties. Therefore, some potential human resource management interventions should be taken. From the perspective of the supervisor, organizations should recognize the importance of restricting and managing supervisors’ behaviors, and encourage supervisors to adopt more active management forms. For example, some policies and regulations can be used to limit negative behaviors of supervisors. At the same time, various actions such as corporate cultural guidance and regular management skills training can be used to improve supervisor’s awareness of the harm of abuse supervision, strengthen the self-control ability of the supervisor, and minimize the possibility of abusive supervision. From the perspective of the subordinate, organization shall pay attention to the changes of the psychological and physical resources of subordinates in a timely manner, maintain constant communication with subordinates, and timely understand the psychological dynamics of employees. For example, the human resources department can organize diversified group building activities to reduce the work pressure of employees and help them release negative emotions, thereby creating a positive work atmosphere, giving full support to subordinates’ work, and strengthening their psychological resources.

### 5.3. Limitations and Future Research

Like previous studies, our studies have some limitations as well, which should be noted. First, compared with Table 4, the proportion of the variance explained in Table 5 is reduced. That means, there still exists many other mechanisms between the relationship of peer abusive supervision and silence. Future research can further study other emotional mechanisms and compare the differences between them based on our research. Second, our data were collected from eight real estate and insurance companies. Future research could try to collect data from other industries, expanding the scope beyond "real estate and insurance companies", which could further verify our model.

Third, in order to avoid violating the assumption of independence in our data and performing a multi-wave study, all of the variables we used were measured by employees’ self-reports. Although the result of the common method variance test is accepted, it is better to design a multi-source study. Future studies could measure the variables from different sources (e.g., supervisor and subordinate) to form a multiple subordinates nested, using multi-level (e.g., hierarchical linear modeling) or random coefficient method to validate our model again. 

Fourth, our research contributes to examine a new mechanism: affect, which potentially underlies the effects of peer abusive supervision. However, we fail to take additional mechanisms into account. Peer abusive supervision has previously been linked to negative outcomes via deontic justice, moral and resources mechanisms, etc., which have been well established [5,69,70,71]. Thus, it is crucial for future research to demonstrate that the indirect relation of peer abusive supervision with silence through the affective mechanism is incremental to deontic justice, moral, and resources mechanisms. Future research can demonstrate this by controlling the above alternative mechanisms when testing the full model, as recent articles published in top journals have done [72,73], which may further develop the affective event theory.

Fifth, affective events theory posits that emotions unfold in real time as people experience affect-laden events, which might include abusive behavior exhibited by supervisors. Affective reactions then precipitate fast-acting affect-based behavior and slow-acting attitude-based behavior [17,18,24]. The present research focuses on the fast-acting affect-based path, exploring the abusive supervision’s negative spillover effects. Future studies should take the slow-acting attitude-based path into account as well. Worth mentioning, it is not only the limitations that our study have but also the gap in affective events theory empirical studies, which ignores the time factors (e.g., fast-acting affect-based path vs. slow-acting attitude-based path). Future research could conduct an experience sampling method study where they measure abusive supervisory behavior, affective reactions, and silence each day for a period of 10−15 workdays [74,75,76]. This would enable ebbs and flows in abusive behavior and the downstream consequences for affect and silence to be captured. Combined with the fourth part of this section, future research may promote the development of affective events theory, which has been at a standstill for a long time.

## 6. Conclusions

From the perspective of third parties, our research investigates the effect of abusive supervision on third parties’ behavior. We propose a theoretical model of implicit behavior response of third parties to peer abusive supervision. Through a longitudinal questionnaire survey of 283 front-line employees, we found that peer abusive supervision has a positive predictive effect on third parties’ silence. Furthermore, their workplace anxiety plays a mediating role in this process. In addition, the high level of core self-evaluation can alleviate the induced effect of peer abusive supervision on workplace anxiety, and further reduce the mediating role of workplace anxiety between peer abusive supervision and silence of third parties. In conclusion, the empirical results show that third parties will evaluate negative work events according to their own situation after they perceive peer abusive supervision. They will then show workplace anxiety, and further implement implicit behavior (silence). Worth mentioning, individuals with a high core self-evaluation can effectively resist such external negative work events. The testing of the above moderated mediation mechanisms helped to fill the gaps, as existing literatures of abusive supervision from third-parties’ perspective mainly focus on third parties’ explicit behavior response to peer abusive supervision, ignoring their implicit reactions (e.g., silence) and the emotional mechanism among it. Moreover, our findings suggest methods that organizations can adopt to resist the negative spillover effects of abusive supervision.

## Figures and Tables

**Table 1:** Demographic information (n = 283).

Feature	Category	Quantity	Percentage
Gender	male	161	0.57
	Female	122	0.43
Age	30 years old and below	162	0.572
	31–40 years old	88	0.311
	Over 40 years old	33	0.117
Education	Senior high school and below	22	0.078
	Training school	27	0.095
	Undergraduate	158	0.558
	Postgraduate and above	76	0.269

**Table 2:** CFA conducted to examine factor structure of the scales used in the study (n = 283).

Model	χ^2^/df	NFI	IFI	CFI	RMR	RMSEA
Four factors: PAS; WA; SL; CSE	2.361	0.938	0 963	0.963	0.067	0.069
Three factors a: PAS+SL; CSE; WA	10372	0.720	0.740	0.739	0.162	0.182
Three factors b: PAS+WA; CSE; SL	9.069	0.755	0.776	0.775	0.197	0.169
Two factors: PAS+WA; CSE+SL	16.225	0.555	0.571	0.569	0.271	0.232
Single factor: PAS+WA+CSE+SL	23.564	0.349	0.359	0.357	0.283	0.283

Note: PAS indicates peer abusive supervision, WA indicates workplace anxiety, SL indicates silence, CSE indicates core self-evaluation; “+” indicates combination of factors.

**Table 3:** Means, standard deviations, and inter-correlations among the study variables. (n = 283).

Variable	M	SD.	1	2	3	4	5	6	7
Gender	1.44	0.50	1						
Age	2.54	0.70	−0.132 *	1					
Education	3.02	0.82	0.007	−0.201 *	1				
PAS	1.69	0.89	−0.118 *	0.055	−0.004	1			
WA	2.31	1.02	−0.002	−0.094	0.097	0.304 **	1		
SL	3.30	1.46	0.074	−0.209 **	0.176 **	0.382 **	0.351 **	1	
CSE	3.27	1.02	0.140 *	−0.045	0.025	−0.003	−0.133 *	−0.153 *	1

Note: PAS indicates peer abusive supervision, WA indicates workplace anxiety, SL indicates silence, CSE indicates core self-evaluation; *: *p* < 0.05, **: *p* < 0.01.

**Table 4:** Regression test of third parties’ silence (n = 283).

Variable	Third Parties’ Silence						
	Model 1	Model 2	Model 3	Model 4	Model 5	Model 6	Model 7
**Gender**	0.050	0.096	0.054	0.091	0.121 *	0.112 **	0.114 *
**Age**	−0.174 *	−0.189 **	−0.147 *	−0.168 **	−0.193 **	−0.174 **	−0.156 **
**Education**	0.141 *	0.139 *	0.116 *	0.123 *	0.143 **	0.128 *	0.133 *
**PAS**		0.402 **		0.333 **	0.406 **	0.344 **	0.330 **
**WA**			0.326 **	0.221 **		0.197 **	0.197 **
**CSE**					−0.182 **	−0.154 **	−0.172 **
**WA × CSE**							−0.109 *
**R^2^**	0.065	0.224	0.169	0.267	0.257	0.290	0.301
**Adjusted R^2^**	0.055	0.213	0.157	0.254	0.243	0.275	0.284
**F**	6.442 **	20.089 **	14.160 **	20.228 **	19.133 **	18.818 **	16.954 **

Note: PAS indicates peer abusive supervision, WA indicates workplace anxiety, CSE indicates core self-evaluation; *: *p* < 0.05, **: *p* < 0.01.

**Table 5:** Regression test of third parties’ workplace anxiety (n = 283).

Variable	Third Parties’ Workplace Anxiety			
	Model8	Model 9	Model 10	Model 11
**Gender**	−0.012	0.024	0.043	0.012
**Age**	−0.083	−0.095	−0.098	−0.097
**Education**	0.077	0.076	0.079	0.080
**PAS**		0.315**	0.318**	0.375**
**CSE**			−0.140 *	−0.147 **
**PAS × CSE**				−0.212 **
**R^2^**	0.015	0.113	0.132	0.173
**Adjusted R^2^**	0.005	0.100	0.117	0.155
**F**	1.436	8.868 **	8.459 **	9.594 **

Note: PAS indicates peer abusive supervision, CSE indicates core self-evaluation; *: *p* < 0.05, **: *p* < 0.01.

**Table 6:** Bootstrap result of mediation effect (n = 283).

**Direct impact of peer abusive supervision on third parties’ silence:**					
Estimate	S.E.	t	p	LLCI	ULCI
0.3010	0.0560	5.3762	0.0000	0.1908	0.4112
**Indirect impact of peer abusive supervision on third parties’ silence:**					
	Estimate	Boot S.E.	Boot LLCI	Boot ULCI	
Third parties’ workplace anxiety	0.0793	0.0346	0.0311	0.1706	

Note: S.E. indicates Standard error; LLCI and ULCI indicate the minimum and maximum values of the confidence interval.

**Table 7:** Bootstrap result of moderated mediation effect (n = 283).

	Conditional Indirect Effect				Moderated Mediator			
	Estimate	S. E.	BC 95% CI		INDEX	S.E.	BC 95% CI	
**Low CSE**	0.1196	0.0391	0.0568	0.2211	−0.0368	0.0138	−0.0615	−0.0061
**Middle CSE**	0.0828	0.0314	0.0334	0.1671				
**High CSE**	0.0461	0.0293	0.0098	0.1288				

Note: CSE indicates core self-evaluation, low CSE represents mean “−1” SD (Standard Deviation), and high CSE represents mean “+1” SD; S.E. indicates Standard Error, BC indicates Biased Corrected, CI indicates Confidence Interval.
